# Design and Numerical Investigation of a Lead-Free Inorganic Layered Double Perovskite Cs_4_CuSb_2_Cl_12_ Nanocrystal Solar Cell by SCAPS-1D

**DOI:** 10.3390/nano11092321

**Published:** 2021-09-07

**Authors:** Yizhou He, Liyifei Xu, Cheng Yang, Xiaowei Guo, Shaorong Li

**Affiliations:** 1 School of Optoelectronic Science and Engineering, University of Electronic Science and Technology of China, Chengdu 610054, China; 201922050416@std.uestc.edu.cn (Y.H.); 201922050415@std.uestc.edu.cn (L.X.); lsrxt@126.com (S.L.); 2 Yangtze Delta Region Institute (Huzhou), University of Electronic Science and Technology of China, Huzhou 313001, China; 3 Key Laboratory of Display Science and Technology of Sichuan Province, University of Electronic Science and Technology of China, Chengdu 610054, China

**Keywords:** lead-free layered double perovskite, Cs_4_CuSb_2_Cl_12_ nanocrystals, solar cell, power conversion efficiency

## Abstract

In the last decade, perovskite solar cells have made a quantum leap in performance with the efficiency increasing from 3.8% to 25%. However, commercial perovskite solar cells have faced a major impediment due to toxicity and stability issues. Therefore, lead-free inorganic perovskites have been investigated in order to find substitute perovskites which can provide a high efficiency similar to lead-based perovskites. In recent studies, as a kind of lead-free inorganic perovskite material, Cs_4_CuSb_2_Cl_12_ has been demonstrated to possess impressive photoelectric properties and excellent environmental stability. Moreover, Cs_4_CuSb_2_Cl_12_ nanocrystals have smaller effective photo-generated carrier masses than bulk Cs_4_CuSb_2_Cl_12_, which provides excellent carrier mobility. To date, there have been no reports about Cs_4_CuSb_2_Cl_12_ nanocrystals used for making solar cells. To explore the potential of Cs_4_CuSb_2_Cl_12_ nanocrystal solar cells, we propose a lead-free perovskite solar cell with the configuration of FTO/ETL/Cs_4_CuSb_2_Cl_12_ nanocrystals/HTL/Au using a solar cell capacitance simulator. Moreover, we numerically investigate the factors that affect the performance of the Cs_4_CuSb_2_Cl_12_ nanocrystal solar cell with the aim of enhancing its performance. By selecting the appropriate hole transport material, electron transport material, thickness of the absorber layer, doping densities, defect density in the absorber, interface defect densities, and working temperature point, we predict that the Cs_4_CuSb_2_Cl_12_ nanocrystal solar cell with the FTO/TiO_2_/Cs_4_CuSb_2_Cl_12_ nanocrystals/Cu_2_O/Au structure can attain a power conversion efficiency of 23.07% at 300 K. Our analysis indicates that Cs_4_CuSb_2_Cl_12_ nanocrystals have great potential as an absorbing layer towards highly efficient lead-free all-inorganic perovskite solar cells.

## 1. Introduction

In the last decade, lead-based perovskite solar cells (PSCs) have witnessed tremendous growth in photovoltaic applications due to their good optical and electrical properties [1]. Typical organic–inorganic hybrid PSC attained power conversion efficiency (PCE) of over 25% by 2020, and include methylammonium lead halide (MAPbX_3_) [2]. Despite these exciting developments, PSCs still face some challenges with respect to commercialization, e.g., their stability, and the toxic nature of lead [3,4,5]. These challenges can be addressed by developing an inorganic lead-free Perovskite absorbing layer [6,7]. Metal cations from the same family as Pb^2+^—Ge^2+^ and Sn^2+^—were first considered to replace Pb^2+^. However, Ge^2+^ and Sn^2+^ are highly susceptible to being oxidized to the tetravalent state (Ge ^4+^, Sn ^4+^) in air [8,9,10]. Subsequently, Bi^3+^ and Sb^3+^ were used as heterovalent substitutes for Pb^2+^ to synthesize two-dimensional or zero-dimensional chalcogenides such as Cs_3_Sb_2_X_9_, Cs_3_Bi_2_X_9_ (X = Cl, Br, I) [11,12,13,14,15]. Recently, double perovskite structure A_2_B’B”X_6_ has been proposed as a promising substitution. This structure is formed by replacing the lead ions in two adjacent lattices with a pair of nontoxic heterovalent (i.e., monovalent and trivalent) metal cations. However, the most typical double perovskite, Cs_2_AgBiBr_6_, is not suitable for photovoltaic (PV) applications due to its wide bandgap of 2.19 eV and the indirect nature of the bandgap [16,17,18].

To further explore lead-free inorganic perovskite candidates suitable for PV applications, Cs_4_CuSb_2_Cl_12_(CCSC) has been proposed. Experiments have demonstrated its impressive photoelectric properties, which include narrow direct bandgap (1.0 eV) and excellent environmental stability (under humidity, heat, and light conditions) [19,20]. However, the bulk CCSC has been inferred to exhibit a high electron effective mass, which results in tardy carrier mobility and consequently hinders its performance in solar cells and other optoelectronic devices [21]. Reduction of the particle size to the nanoscale has been widely demonstrated to be an effective strategy for tuning the energy band structure of materials in accordance with the quantum confinement effect [22,23,24]. In 2019, Kuang et al. fabricated CCSC nanocrystals (NCs) with an average particle size of ~3 nm using a top-down ultrasonic exfoliation technique and subsequently fabricated CCSCNC thin film (500nm) by centrifugal casting of the CCSCNC solution onto FTO glass, using oleic acid (OA) as the organic ligand [25]. The resulting NCs possessed a direct bandgap of 1.6 eV and low effective photo-generated carrier masses. In addition, CCSCNCs have been demonstrated to have excellent environmental stability (under heat, humidity, and light conditions). In 2020, Tong et al. fabricated a thin-film-based high-speed photodetector by casting high-concentration CCSCNC hexane solution, using OA, oleylamine (OAm), and 1-octadecene (ODE) as the ligands on a quartz substrate [26]. The excellent carrier mobility in the CCSCNCs were demonstrated using a high-speed photodetector, suggesting that CCSCNCs have great potential as the absorber layer for solar cells. In 2021, Ashitha P. et al. proved that CCSCNCs (~3.9 nm) can efficiently catalyze the ferricyanide reduction and dye degradation reactions as a photocatalyst, and CCSCNCs have strong absorption throughout the visible region [27]. NCs, as particles with one or more dimensions less than 100 nanometers (nm), have different properties from their bulk materials due to the quantum-confinement effect [28]. In particular, perovskite NCs are considered ideal candidates for next-generation photovoltaic applications due to their high electrical conductivity, broad absorption spectrum, variable band gap, and structural compatibility [28,29]. In recent years, perovskite NCs-based solar cells have developed rapidly, and the highest efficiency has reached 16.6% [30].

To the best of our knowledge, there are no reports on CCSCNCs for solar cells. In this paper, to explore the potential of CCSCNCs in solar cells, we propose a CCSCNC PSC with a structure involving a fluorine-doped tin oxide (FTO)/electron transport layer (ETL)/CCSCNCs/hole transport layer (HTL)/Au; then, by selecting a suitable hole transport material (HTM), electron transport material (ETM), thickness of the absorber layer, doping densities, defect density in the absorber, interface defect densities, and working temperature point, we predict that the CCSCNC solar cell with the FTO/TiO_2_/CCSCNCs/Cu_2_O/Au structure can attain a PCE of 23.07% at 300 K. In addition, we investigate the factors affecting the performance of the CCSCNCs solar cell for enhancing its performance, providing a guide for future experiments.

## 2. Device Structure and Simulation Parameters

### 2.1. Numerical Method

The numerical simulation software used in this work is solar cell capacitance simulator (SCAPS-1D 3.3.10), a one-dimension solar cell simulation software program developed by investigators at Ghent University [31]. Fundamentally, SCAPS-1D executes three sets of PV equations for the hole and electron carrier densities, respectively. These three equations are shown below:

(1) $$\begin{array}{l} \frac{d}{dx}[\varepsilon (x)\frac{d\psi }{dx}]=q[p(x)-n(x)+N_{d}(x)-N_{a}(x)+p_{t}(x)-n_{t}(x)] \end{array}$$

(2) $$-\frac{1}{q}\frac{dJ_{n}}{dx}+R_{n}(x)-G(x)=0$$

(3) $$-\frac{1}{q}\frac{dJ_{p}}{dx}+R_{p}(x)-G(x)=0$$

where *x* denotes the coordinate position; *ψ* and *ε* represent the electrostatic potential and the relative permittivity; *n* denotes the number of electrons; similarly, *p* denotes the number of holes; *N_d_* represents the ionized donor concentration; *N_a_* represents the ionized acceptor density; *n_t_* is the number of trapped electrons; *p_t_* is the number of trapped holes; *J_n_* represents the current density of electrons; *J_p_* represents the current density of holes; *G*(*x*) is the photo-generated rate; the recombination rate of electrons is represented by *R_n_*(*x*); and the recombination rate of holes is represented by *R_p_*(*x*).

The optical absorption constant *α* of the CCSCNCs is calculated from the following equation of the optical absorption model Equation [31]:

(4) $$\alpha \left(\lambda \right)=\left(A+\frac{B}{hv}\right){\left(hv-E_{g}\right)}^{\frac{1}{2}}$$

where *A* and *B* are the model parameters, and *E_g_* is the actual band gap of the material. The monochromatic photon in this work we set as 10^18^ s^−1^. The shunt resistance of the CCSCNCs solar cell is 4200 Ω∙cm^2^. As for the series resistance of the device, we set it as 1 Ω∙cm^2^. For working condition, we choose a standard AM 1.5G illumination and a continuous temperature 300 K [28].

### 2.2. Device Structure and Materials

**Figure 1:**
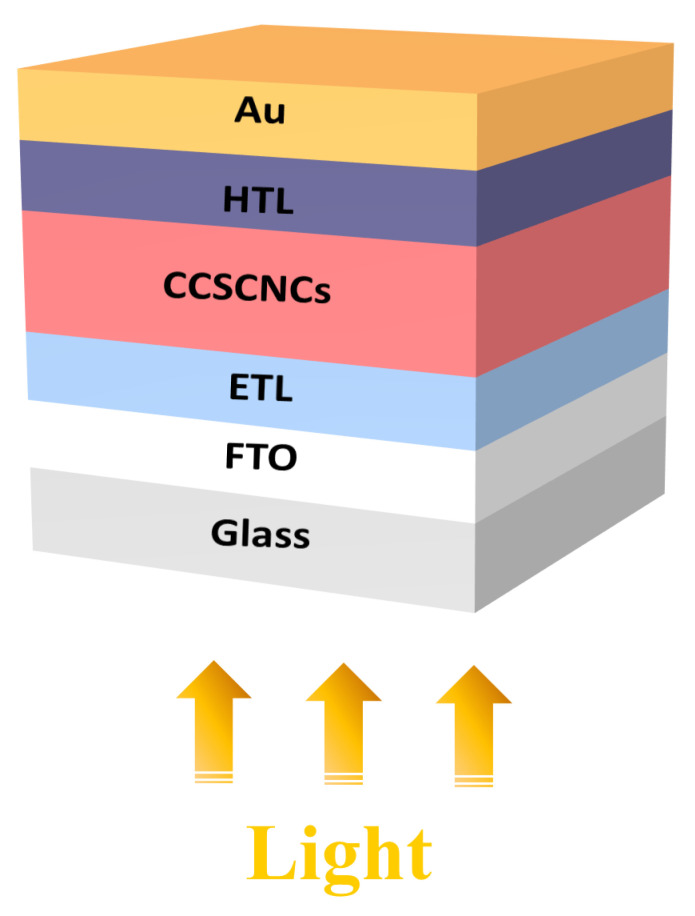
Schematic of the CCSCNCs-based perovskite solar cell.

The PSC structure of FTO/ETL/CCSCNCs/HTL/Au is schematically shown in Figure 1. In the structure, lead-free inorganic CCSCNCs were used as the absorber layer. The ETM and HTL will be carefully chosen for the purpose of high efficiency. The ETL is chosen from often-used materials in PSCs, e.g., TiO_2_, PCBM, ZnO, and IGZO. Similarly, the HTL is also chosen from often used materials in PSCs, e.g., P3HT, PEDOT:PSS, Spiro-OMETAD, Cu_2_O, and CuI. Table 1 and Table 2 include the basic parameters of the ETL, HTL and CCSCNCs, which were extracted from the previously published literature [19,20,21,25,26,32,33,34,35,36,37,38,39,40]. The bulk defect parameters in the absorber layer and the interface defect parameters at ETL/Absorber and Absorber/HTL interfaces are presented in Table 3.

## 3. Results and Discussion

We investigate the crucial factors affecting performance on the CCSCNCs solar cell and select the appropriate parameters to improve its performance. Firstly, we determine the suitable hole transport layer materials (HTMs) and electron transport materials (ETMs) for our CCSCNC PSC. Next, we optimize the absorber layer thickness to enhance the performance of the device. Finally, we investigate the effect of doping density in the transport layers, the effect of bulk defect in the absorber layer, the effect of interface defect densities at the CCSCNCs/ETL and CCSCNCs/ETL interfaces, and the effect of operating temperature. In addition, we select the appropriate values of the three parameters to enhance the performance of the device step by step. At each step of the optimization process, the other parameters are considered constants, and only the parameters to be optimized are varied. Each single optimization step is performed based on the completion of the previous optimizations.

### 3.1. HTMs Selection

**Figure 2:**
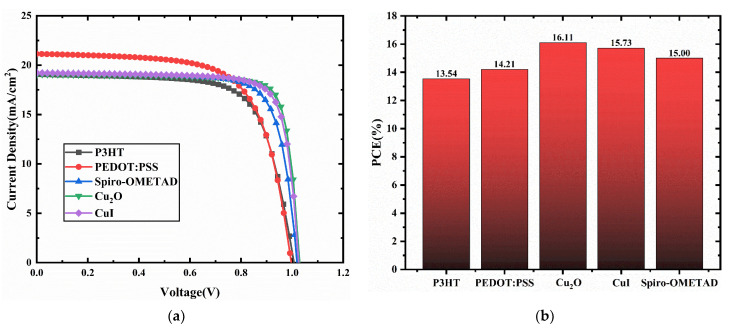
(**a**) Comparison of J-V characteristic curves for different HTMs as HTL; (**b**) PCE for different HTMs as HTL.

**Figure 3:**
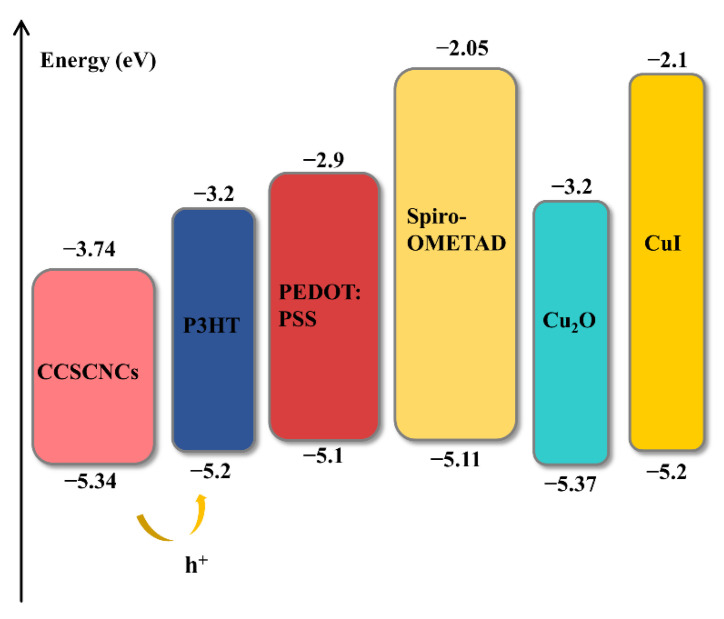
Band alignment between HTL materials and CCSCNCs.

In this selection, the HTL/CCSCNCs/TiO_2_/Au structure is used to select the most appropriate HTM. Figure 2 presents the J-V plots and PCE statistical graph of the device with different HTMs. The detailed performance parameters of the cells with different HTMs are shown in Table 4. The band alignment between CCSCNCs and HTMs is shown in Figure 3. The valence band offset (*VBO*) is defined by the following Equation (5), which denotes the difference between valence band (*VB*) level of HTL and that of the perovskite.

(5) $$VBO=E_{V}\left(HTL\right)-E_{V}\left(Absorber\;Layer\right)$$

where *Ev* (*HTL*) denotes the *VB* level of *HTL*, *Ev* (*Absorber Layer*) denotes the *VB* level of the absorber layer. When the *VB* energy level of the CCSCNCs absorber layer is lower than that of HTL, the *VBO* becomes negative. It leads to the formation of an energy cliff between the absorber and HTL. The energy cliff can promote holes transporting *HTL* from the absorber layer. However, it is not the case that the larger the energy cliff is, the better it is. The difference between the absolute value of the band gap of the absorber layer and that of *VBO* represents the activation energy for carrier recombination, and a smaller activation energy means that the carriers are more likely to recombine [41]. Therefore, a large energy cliff will cause the CCSCNCs/Cu_2_O interface have a smaller carrier activation energy, resulting in enhanced recombination at the interface [42]. This can deteriorate the performance of the device. From Figure 3, it can be observed that the *VBO*s are −0.14 eV (P3HT), −0.24 eV(PEDOT:PSS), −0.23 eV(Spiro-OMETAD), +0.03 eV(Cu_2_O), −0.24 eV(CuI) respectively. The *VBO*s of all materials are negative, except for the *VBO* of Cu_2_O, which is positive. This indicates that all other materials form energy cliffs with the absorber layer and do not block hole transport. A positive *VB* for Cu_2_O means that an energy spike forms between the Cu_2_O and the absorber layer. The formation of this energy spike will hinder hole transport. In particular, small spikes have little effect on the hindrance of hole transport but can increase the activation energy of the interfacial recombination. This leads to a reduction of the interfacial recombination and thus to an improvement of the device performance. As shown in Figure 2, Cu_2_O used as HTM has a larger open-circuit voltage (Voc) and fill factor (FF) compared to other HTMs, because Cu_2_O has the smallest activation energy of the interfacial recombination. Therefore, the device used Cu_2_O, as the HTM possesses the highest PCE. Therefore, we obtain the optimal calculation result of the device by setting Cu_2_O as the HTM, which has a Voc of 1.03 V, a short circuit current density (Jsc) of 19.07 mA/cm^2^, an FF of 82%, and a PCE of 16.11%.

### 3.2. ETMs Selection

**Figure 4:**
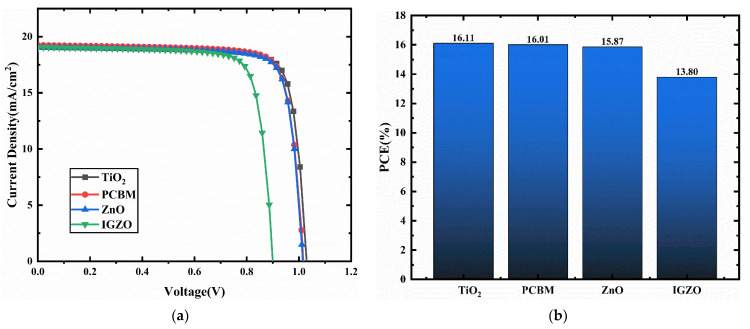
(**a**) Comparison of the J-V characteristic curves for different HTMs as HTL; (**b**) PCE for different ETMs as ETL.

**Figure 5:**
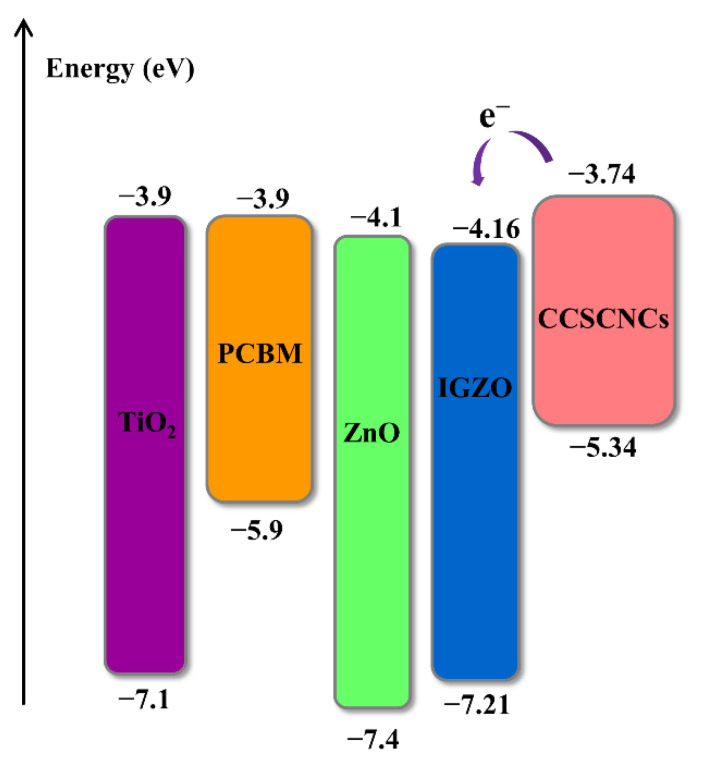
Band alignment between ETL materials and CCSCNCs.

In this selection, the Cu_2_O/CCSCNCs/ETL/Au structure is used to select the most appropriate ETM. Figure 4 presents the J-V plots and PCE statistical graph of the device with different ETMs. The detailed performance parameters of the cells with different ETMs are shown in Table 5. The band alignment between CCSCNCs and ETMs is shown in Figure 5. The conduct band offset (*CBO*) is defined by the following Equation (6), which denotes the difference between conduct band (*CB*) level of *ETL* and that of the perovskite.

(6) $$CBO=E_{C}\left(Absorber\;Layer\right)-E_{C}\left(ETL\right)$$

where *Ec* (*Absorber Layer*) denotes the *CB* level of the absorber layer, *Ev* (*ETL*) denotes the *CB* level of ETL. When the energy level of HTL is lower than that of the CCSCNCs absorber layer, the *CBO* becomes negative. The negative *CBO* is beneficial for electron transport to ETL from the absorber layer due to the formation of the energy cliffs between ETL and the absorber layer. Similarly, a small cliff is beneficial, but a large cliff enhances the interfacial recombination, resulting in the deterioration of performance [41,42]. From Figure 4, it can be observed that the TiO_2_ used as ETM has the highest PCE compared to other ETMs. Figure 5 shows that the *CBO*s are −0.16 eV(TiO_2_), −0.16 eV(PCBM), −0.36 eV(ZnO), and −0.42 eV(IGZO), respectively. Both TiO_2_ and PCBM have the smallest absolute *CBO* values, which are smaller than those of the other two materials. TiO_2_ used as ETM has a slightly higher PCE than PCBM because the dielectric constant of TiO_2_(ε_r_ = 9) is higher than that of PCBM(ε_r_ = 3.9), which is consistent with the previous literature [43]. Therefore, we select TiO_2_ as the most appropriate ETM. The device with TiO_2_ ETL has the Voc of 1.03 V, Jsc of 19.07 mA/cm^2^, FF of 82%, and PCE of 16.11%.

### 3.3. CCSCNC Thickness

**Figure 6:**
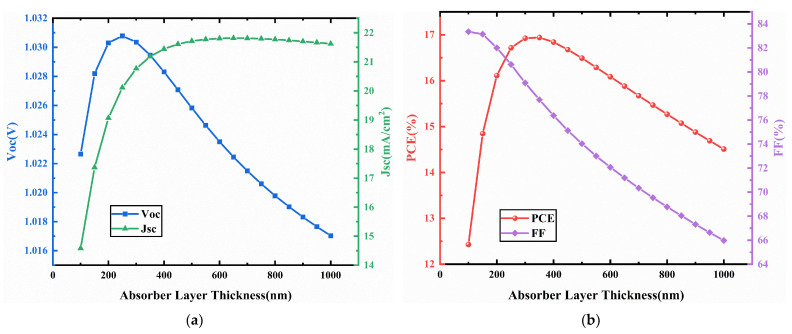
(**a**) Change in Voc and Jsc against CCSCNC absorber layer thickness variation; (**b**) change in PCE and FF against CCSCNC absorber layer thickness variation.

Since the thicknesses of HTL and ETL have very small effects on the device performance, we only optimize the thickness of the absorber layer, which plays a decisive role in the device performance. The thickness of the CCSCNC layer is optimized within the range of 100 nm to 1000 nm. From Figure 6, it can be seen that the Voc increases with increasing absorber layer thickness and reaches a maximum value of 1.031 V at 250 nm, which should be attributed to the greater number of generated electrons and holes. However, above 250 nm, the Voc decreases due to the fact that excess absorption of photons may enhance the heat production in the device [44].

With increasing absorber layer thickness, the Jsc increases quickly, and above 400 nm, it continues to increase, but only slightly. This trend can be attributed to the fact that a thick absorber layer does not create more carriers [45]. It is noted that, as the absorber layer thickness increases, the FF decreases quickly, which is due to the increase in series resistance of the absorber layer. As a result, the PCE firstly increases, and then reaches a maximum value at 350 nm, after which the PCE gradually decreases.

The best performance occurs at a thickness of 350 nm, with Voc = 1.03 V, Jsc = 21.18 mA/cm^2^, FF = 77.69%, PCE = 16.94%.

### 3.4. Effect of the Doping Density in the Transport Layers

To understand the effect of doping in the HTL and ETL on device performance, we vary the doping density from 10^16^ cm^−3^ to 10^20^ cm^−3^.

**Figure 7:**
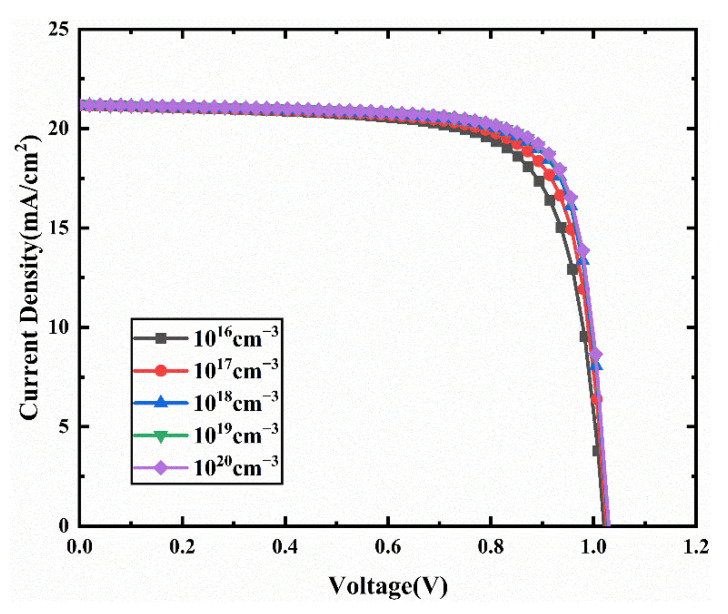
Comparison of J-V characteristic curves for different acceptor density (N_A_).

**Figure 8:**
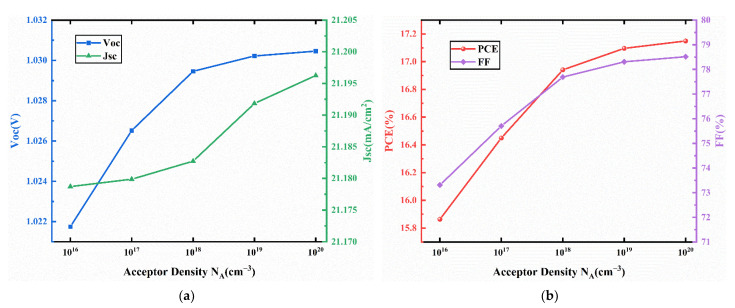
(**a**) Change in Voc and Jsc against acceptor density (N_A_) variation; (**b**) change in PCE and FF against HTL doping density (N_A_) variation.

J-V characteristics curves for different acceptor density in HTL are presented in Figure 7. The corresponding device performances are presented in Figure 8. In Figure 8, Voc, Jsc, FF increase with increasing acceptor density. The increase of the acceptor density in the HTL can increase the hole mobility and charge density, leading to a reduction in the resistivity of the HTL, resulting in increased Jsc and FF [46,47]. In addition, the increase of HTL doping density leads to the enhancement of the interfacial electric field between HTM and ETM, which increases the potential used to separate excitons and decreases the recombination rate [48]. Thus, a better extraction of electrons and holes from the absorber layer can be achieved, which improves the Voc. Therefore, the PCE increases from 15.86% to 17.15% when the acceptor density increases from 10^16^ cm^−3^ to 10^20^ cm^−3^. Therefore, we select an acceptor density N_A_ of 10^20^ cm^−3^, and the performance parameters of the device with this acceptor density are an FF of 78.52%, Jsc of 21.20 mA/cm2, Voc of 1.03 V, and PCE of 17.15%.

**Figure 9:**
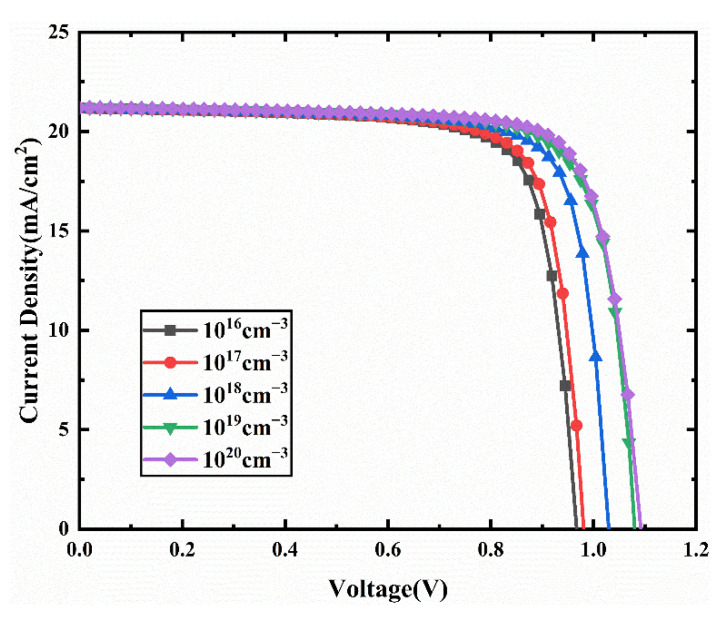
Comparison of J-V characteristic curves for different donor density (N_D_).

**Figure 10:**
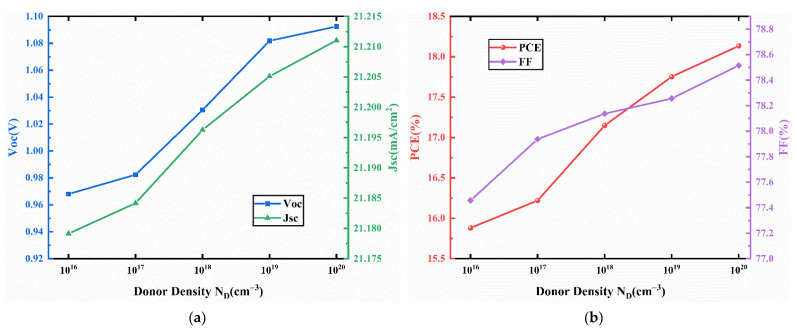
(**a**) Change in Voc and Jsc against ETL donor density (N_D_) variation; (**b**) change in PCE and FF against ETL doping density (N_D_) variation.

Similarly, J-V characteristics curves for different donor density in ETL are presented in Figure 9. The corresponding device performances are presented in Figure 10. Figure 10 shows that Voc, Jsc, and FF increase with the increase of donor density. Similar to the doping density of HTL discussed above, the interfacial electric field between ETL and HTL is enhanced as the doping density of ETL increases, which contributes to the separation of excitons and reduces the recombination [48]. The PCE increases from 15.88% to 18.13% with an increase in donor density from 10^16^ cm^−3^ to 10^20^ cm^−3^. Therefore, we select a donor density N_D_ of 10^20^ cm^−3^, and the performance parameters of the device with this donor density are an FF of 78.52%, Jsc of 21.21 mA/cm^2^, Voc of 1.09 V, and PCE of 18.13%.

### 3.5. Effect of the Bulk Defect Density

**Figure 11:**
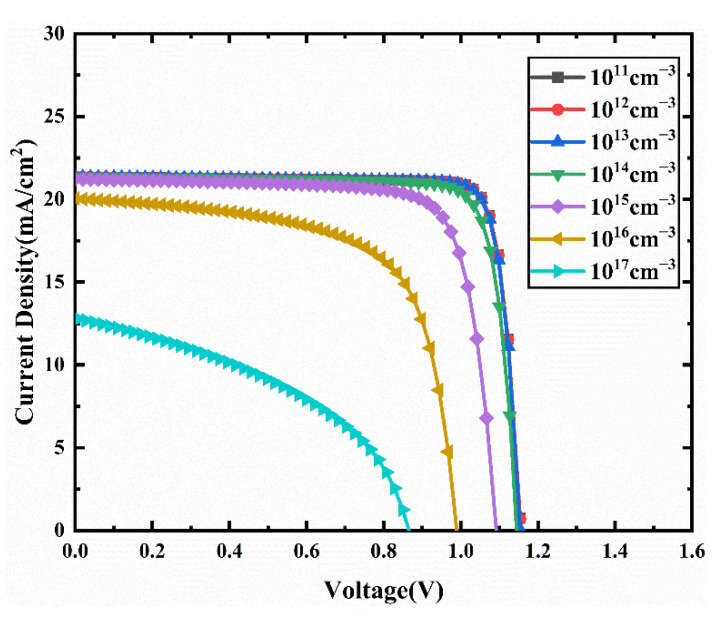
Comparison of J-V characteristic curves for different bulk defect density.

**Figure 12:**
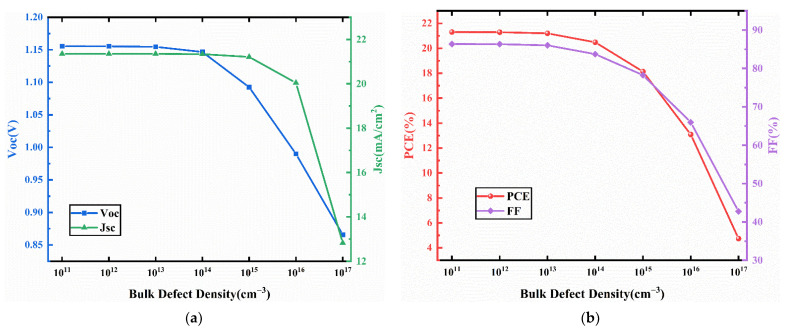
(**a**) Change in Voc and Jsc against absorber layer bulk defect density variation; (**b**) change in PCE and FF against absorber layer bulk defect density variation.

To analyze the effect of the bulk defect density at the interface of the absorber/transport layer on device performance, we vary the bulk defect density in the CCSCNC absorber layer from 10^11^ cm^−3^ to 10^17^ cm^−3^. The J-V characteristics curves for different bulk defect density in the absorber layer are presented in Figure 11. The corresponding device performance is presented in Figure 12. From Figure 12, it can be seen that Voc, Jsc, and FF decrease with increasing bulk defect density due to the fact that the generated electrons and holes are more easily captured by bulk defects [43]. The PCE falls from 21.3% to 4.74% when the bulk defect density increases from 10^11^ cm^−3^ to 10^17^ cm^−3^. When the bulk defect density is below 10^12^ cm^−3^, the effect of the defect density on the PCE becomes weak. Defects are inevitable in the actual perovskite absorber layer, so the device has relatively good performance at a defect density of less than 10^12^ cm^−3^. We select an optimized bulk defect density of 10^12^ cm^−3^, with an obtained Voc of 1.16 V, Jsc of 21.35 mA/cm^2^, FF of 86.33%, and PCE of 21.3%.

### 3.6. Effect of Interface Defect Density

For the interface between the perovskite absorber layer and the transport layer, it has been shown that the interface defects density increases under light, oxygen, humidity, and high temperature, thus degrading the performance of the device. Therefore, it is significant to study the effect of interface defect density on device performance [49].

**Figure 13:**
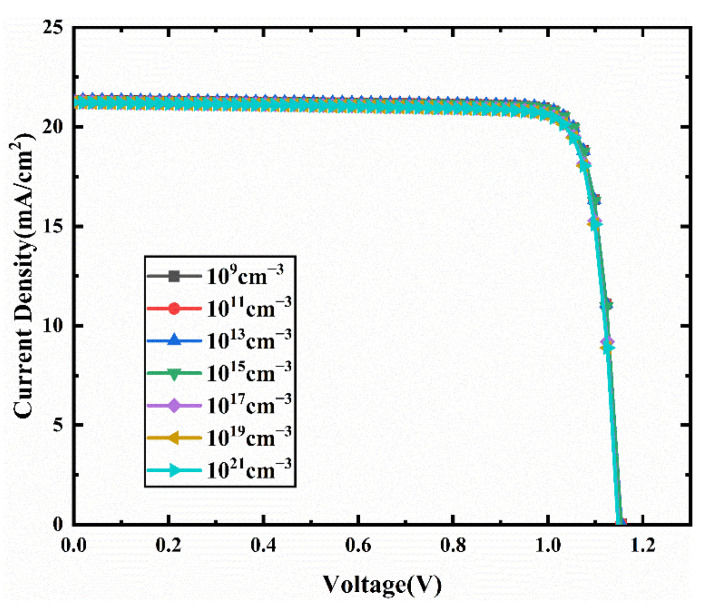
Comparison of J-V characteristic curves for different Cu_2_O/CCSCNCs interface layer defect density.

**Figure 14:**
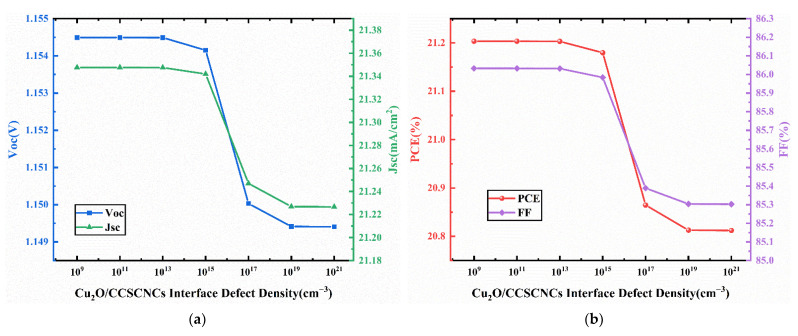
(**a**) Change in Voc and Jsc against Cu_2_O/CCSCNCs interface defect density variation; (**b**) change in PCE and FF against Cu_2_O/CCSCNCs interface defect density variation.

We vary the interface defect density at Cu_2_O/CCSCNCs interface and CCSCNCs/TiO_2_ interface from 10^9^ cm^−3^ to 10^21^ cm^−3^. Figure 13 presents the J-V curves for different interface defect densities in Cu_2_O/CCSCNCs interface layer. Figure 14 presents the corresponding device performance. It shows that Voc, Jsc, and FF decrease with the Cu_2_O/CCSCNCs interface defect density. This trend is attributed to the fact that the higher defect density at Cu_2_O/CCSCNCs interface brings more traps and recombination centers, which results in deteriorating performance of the cells [43]. Therefore, the PCE decreases from 21.20% to 20.81% with the Cu_2_O/CCSCNCs interface defect density increasing from 10^19^ cm^−3^ to 10^21^ cm^−3^. It is obvious that when the defect density at Cu_2_O/CCSCNCs interface layer is below 10^13^ cm^−3^, the effect of the defect density on the PCE becomes weak. Considering that defects are inevitable in the actual interface, relatively good performance can be obtained when the defect density is less than 10^13^ cm^−3^. Thus, we choose 10^13^ cm^−3^ as the defect density at Cu_2_O/CCSCNCs interface layer, with an obtained Voc of 1.16 V, a Jsc of 21.35 mA/cm^2^, an FF of 86.33%, and a PCE of 21.3%.

**Figure 15:**
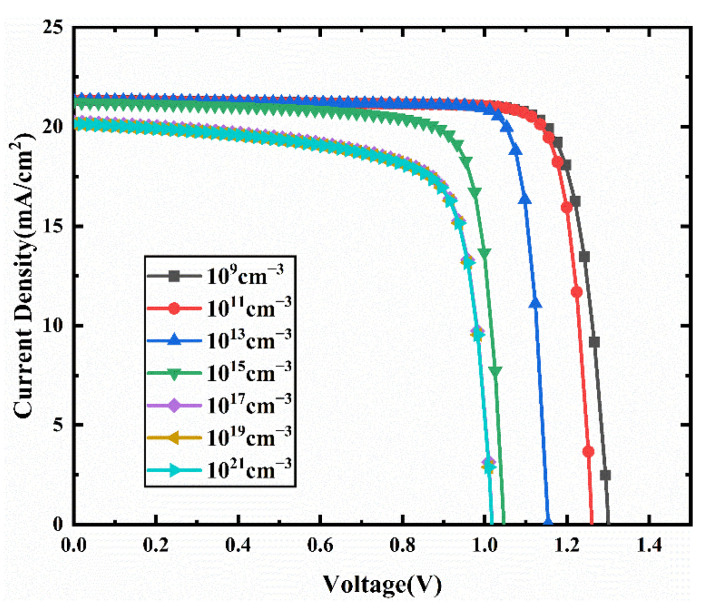
Comparison of J-V characteristic curves for different CCSCNCs/TiO_2_ interface layer defect density.

**Figure 16:**
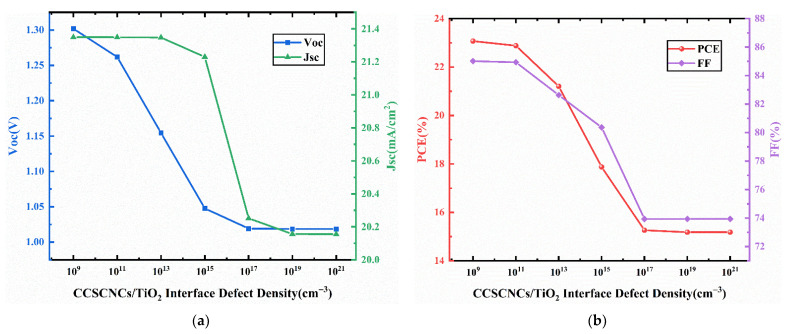
(**a**) Change in Voc and Jsc against CCSCNCs/TiO_2_ interface defect density variation; (**b**) change in PCE and FF against CCSCNCs/TiO_2_ interface defect density variation.

Figure 15 presents the J-V curves for different interface defect density in the CCSCNCs/TiO_2_ interface layer. Figure 16 presents the corresponding device performance. In Figure 16, similar to the CCSCNCs/TiO_2_ interface, Voc, Jsc, and FF decrease with the CCSCNCs/TiO_2_ interface defect density due to the higher defect density at the CCSCNCs/TiO_2_ interface resulting in more traps and recombination centers. Therefore, the PCE decreases from 23.07% to 15.18% with an increase in CCSCNCs/TiO_2_ interface defect density from 10^9^ cm^−3^ to 10^21^ cm^−3^. It is noted that the efficiency reduction due to the increase of CCSCNCs/TiO_2_ interface defect density is much larger than that due to the increase of Cu_2_O/CCSCNCs interface defect density. Obviously, the defect density at the CCSCNCs/TiO_2_ interface has a remarkable influence on the device performance. This is because the number of electron–hole pairs generated at CCSCNCs/TiO_2_ is 10 times higher than that at Cu_2_O/CCSCNCs under light illumination [44]. The higher excess carrier density present at the CCSCNCs/TiO_2_ interface leads to a higher recombination rate. The difference between the two interfaces we obtained is consistent with the previous literature [50,51]. Considering that defects are inevitable in the actual interface, relatively good performance can be obtained when the defect density is less than 10^9^ cm^−3^. Therefore, when the interface defect density at the CCSCNCs/TiO_2_ interface is 10^9^ cm^−3^, the maximum PCE is 23.07%, while FF = 83.02%, Jsc = 21.35 mA/cm^2^, and Voc = 1.3 V.

### 3.7. Effect of Operating Temperature

**Figure 17:**
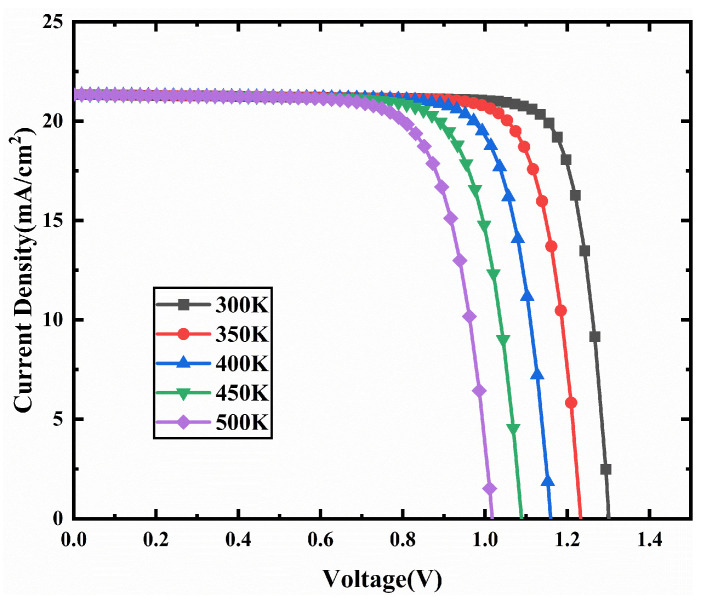
J-V characteristic curves of the device for different operating temperatures.

The actual operating temperature of PSC typically exceeds 80 °C (353 K), and the performance of PSC is highly dependent on the operating temperature [52]. We investigate the effect of operating temperature on the device performance by varying the temperature from 300 K to 500 K. Figure 17 shows that the device performance continues to deteriorate with increasing temperature. As the temperature increases from 300 K to 500 K, the PCE of the device decreases from 23.07% to 16.12%. This phenomenon can be explained by the following Equation (7):

(7) $$\frac{dV_{oc}}{dT}=\frac{\left(V_{oc}-\frac{E_{g}}{q}\right)}{T}$$

where *T* denotes the working temperature, *q* represents the elementary charge, *E_g_* denotes the band gap. As the temperature increases, the Voc decreases, resulting in a lower PCE of the device [53]. In addition, as the temperature increases, the device defect density in the device increases, and the carrier mobility decrease, which deteriorates the device performance [54]. Therefore, we obtain an optimal PCE of 23.07% on the device at 300 K.

### 3.8. Performance with the Optimized Device Structure

**Figure 18:**
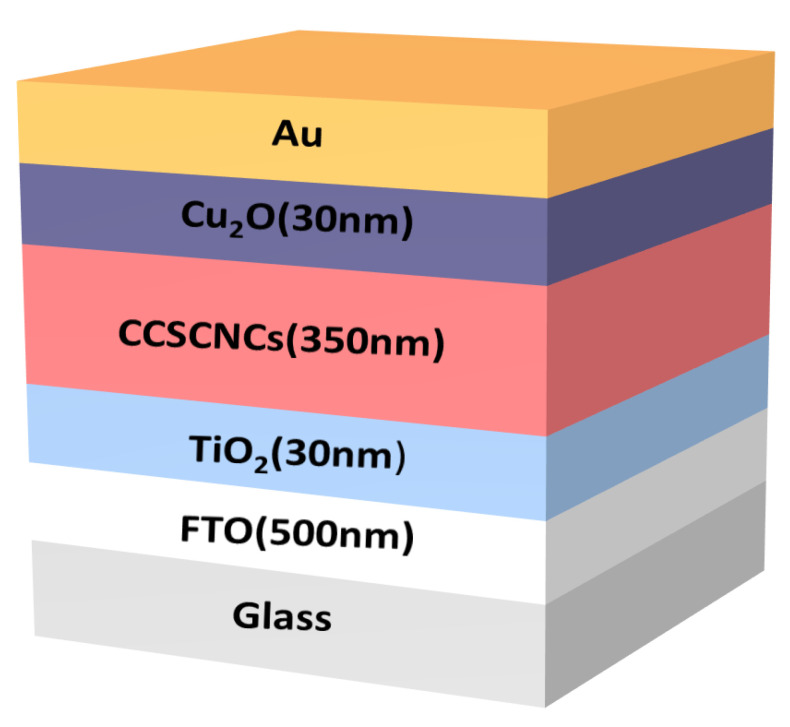
Schematic of the optimized device with FTO/TiO_2_/CCSCNCs/Cu_2_O/Au structure.

**Figure 19:**
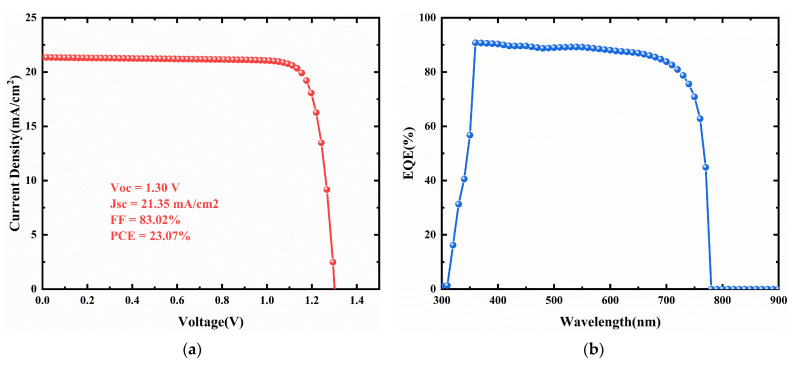
(**a**) J-V curve for the optimized device with FTO/TiO_2_/CCSCNCs/Cu_2_O/Au structure; (**b**) EQE curve for the optimized device with FTO/TiO_2_/CCSCNCs/Cu_2_O/Au structure.

The final optimal device structure is FTO/TiO_2_/CCSCNCs/Cu_2_O/Au, and the thickness of the CCSCNC absorber layer is 350 nm, as shown in Figure 18. The J-V curve for the optimal CCSCNC cell structure is presented in Figure 19a, where the acceptor density in HTL and the donor density in ETL are both 10^20^ cm^−3^, and the bulk defect density in the CCSCNC absorber layer, the interface defect density at Cu_2_O/CCSCNCs interface, and the interface defect density at CCSCNCs/TiO_2_ interface are 10^12^ cm^−3^, 10^13^ cm^−3^, and 10^9^ cm^−3^, respectively. The performance of the optimized device is predicted to have a PCE of 23.07%, Voc of 1.3 V, Jsc = 21.35 mA/cm^2^ and FF = 83.02%.

The external quantum efficiency (EQE) was also calculated. The EQE takes optical performance of the solar cell along with the ratio of charge generation with respect to incident light photons. From Figure 19b, it can be seen that 80~90% quantum efficiency is obtained in the wavelength range of 360 nm–720 nm.

Table 6 provides a performance comparison among various works on lead-free PSC. It shows that different lead-free perovskite absorbers produce different PCEs, e.g., from 13.57% to 27.43%. The corresponding Voc, Jsc, FF values are in the range of 0.8 V~1.9 V, 19.88 mA/cm^2^~40.05 mA/cm^2^, and 35.95%~87.79%, respectively. Although the values for Voc, Jsc, FF we obtained are not the highest among those lead-free PSCs, the PCE is very high. Our simulation results show that the PSC with structure of FTO/TiO_2_/CCSCNCs/Cu_2_O/Au has an exciting PCE of 23.07%. It indicates that the CCSCNCs are very suitable for the absorber layer in PSC.

## 4. Conclusions

In this work, we numerically explored the performance of the Cs_4_CuSb_2_Cl_12_ nanocrystal solar cell using SCAPS-1D. By selecting the appropriate hole transport material, electron transport material, thickness of the absorber layer, doping densities, defect density in the absorber, interface defect densities, and working temperature point, we predicted that the CCSCNCs solar cell with the FTO/TiO_2_/CCSCNCs/Cu_2_O/Au structure could attain a PCE of 23.07% at 300 K. Very high electrical parameters were obtained, with a Jsc of 21.35 mA/cm^2^, a Voc of 1.30 V, an FF of 83.02%, and an external quantum efficiency of 80~90% in the range of 360~720 nm. These exciting results suggest that CCSCNCs could play a momentous role as an absorbing perovskite in achieving a highly efficient lead-free inorganic perovskite solar cell technology. In addition, we investigated the factors affecting the performance of CCSCNCs solar cells in order to enhance their performance, thus providing a guide for future experiments.

## Figures and Tables

**Table 1:** Input parameters for CCSCNCs absorber layer and HTL of the PSC structure.

Parameters	CCSCNCs	P3HT	PEDOT:PSS	Spiro-OMETAD	Cu_2_O	CuI
Thickness, *d* (nm)	200	30	30	30	30	30
Band gap, *E_g_* (eV)	1.6	2	2.2	3.06	2.17	3.1
Electron affinity, *χ* (eV)	3.74	3.2	2.9	2.05	3.2	2.1
Permittivity, ε_r_	10	3	3	3	7.1	6.5
Effective density states at CB, *N_C_* (cm^−3^)	4.5 × 10^18^	1 × 10^20^	2.2 × 10^15^	2.8 × 10^19^	2.5 × 10^18^	2.2 × 10^19^
Effective density states at VB, *N_V_* (cm^−3^)	1.6 × 10^18^	1 × 10^20^	1.8 × 10^18^	1 × 10^19^	1.8 × 10^19^	1.8 × 10^19^
Electron mobility, *μ_n_* (cm^−2^/V·s)	2.5	0.0001	10	1× 10^−4^	200	100
Hole mobility, *μ_p_* (cm^−2^/V·s)	2.5	0.0001	10	2× 10^−4^	80	43.9
Density of n-type doping, *N_D_* (cm^−3^)	1 × 10^13^	0	0	0	0	0
Density of p-type doping, *N_A_* (cm^−3^)	1 × 10^13^	1 × 10^16^	3.17 × 10^14^	1 × 10^18^	9.1 × 10^21^	1 × 10^18^
Defect density, *N_t_* (cm^−3^)	1 × 10^15^	1 × 10^15^	1 × 10^14^	1 × 10^14^	1 × 10^14^	1 × 10^14^
electron thermal velocity (cm/s)	1 × 10^7^	1 × 10^7^	1 × 10^7^	1 × 10^7^	1 × 10^7^	1 × 10^7^
hole thermal velocity (cm/s)	1 × 10^7^	1 × 10^7^	1 × 10^7^	1 × 10^7^	1 × 10^7^	1 × 10^7^
Capture cross-section electrons (cm^2^)	1 × 10^−14^	1 × 10^−15^	1 × 10^−15^	1 × 10^−15^	1 × 10^−15^	1 × 10^−15^
Capture cross-section holes (cm^2^)	1 × 10^−14^	1 × 10^−15^	1 × 10^−15^	1 × 10^−15^	1 × 10^−15^	1 × 10^−15^
Reference	[19,20,21,25,26,32,33,34,37,40]	[33]	[35]	[36]	[34]	[38]

**Table 2:** Input parameters for ETL of the PSC structure.

Parameters	TiO_2_	PCBM	ZnO	IGZO
Thickness, *d* (nm)	30	30	30	30
Band gap, *E_g_* (eV)	3.2	2	3.3	3.05
Electron affinity, *χ* (eV)	4.1	3.9	4.1	4.16
Permittivity, ε_r_	9	3.9	9	10
Effective density states at CB *N_C_* (cm^−3^)	2.2 × 10^18^	2.5 × 10^21^	4 × 10^18^	5 × 10^18^
Effective density states at VB *N_V_* (cm^−3^)	1 × 10^19^	2.5 × 10^21^	1 × 10^19^	1 × 10^18^
Electron mobility, *μ_n_* (cm^−2^/V·s)	20	0.2	100	15
Hole mobility, *μ_p_* (cm^−2^/V·s)	10	0.2	25	0.2
Density of n-type doping, *N_D_* (cm^−3^)	1 × 10^18^	2.93 × 10^17^	1 × 10^18^	1 × 10^17^
Density of p-type doping, *N_A_* (cm^−3^)	0	0	1 × 10^5^	0
Defect density, *N_t_* (cm^−3^)	1 × 10^15^	1 × 10^15^	2 × 10^17^	1 × 10^15^
electron thermal velocity (cm/s)	1 × 10^7^	1 × 10^7^	1 × 10^7^	1 × 10^7^
hole thermal velocity (cm/s)	1 × 10^7^	1 × 10^7^	1 × 10^7^	1 × 10^7^
Capture cross-section electrons (cm^2^)	2 × 10^−^^14^	1 × 10^−^^15^	1 × 10^−^^15^	2 × 10^−^^14^
Capture cross-section holes (cm^2^)	2 × 10^−^^14^	1 × 10^−^^15^	1 × 10^−^^15^	2 × 10^−^^14^
Reference	[33]	[38]	[39]	[37]

**Table 3:** Input parameters of defect inside the absorber and interface defect layers.

Parameters	ETL/Absorber	Absorber/HTL	CCSCNCs
Defect type	Neutral	Neutral	Neutral
Capture cross-section for electrons (cm^2^)	1 × 10^−19^	1 × 10^−18^	1 × 10^−15^
Capture cross-section for holes (cm^2^)	1 × 10^−18^	1 × 10^−19^	1 × 10^−15^
Energetic distribution	Gaussian	Gaussian	Gaussian
Energy level with respect to *E_v_*	0.6	0.6	0.6
Characteristic energy (eV)	0.1	0.1	0.1
Total density (cm^−3^)	1 × 10^13^	1 × 10^13^	1 × 10^15^
Reference	[33]	[33]	[33,40]

**Table 4:** Performance parameters of different HTMs as HTL.

Hole Transport Material	Voc (V)	Jsc (mA/cm^2^)	FF (%)	PCE (%)
P3HT	1.01	19.07	70.47	13.54
PEDOT:PSS	1.00	21.14	67.44	14.21
Spiro-OMETAD	1.02	19.21	76.54	15.00
Cu_2_O	1.03	19.07	82.01	16.11
CuI	1.03	19.22	79.66	15.73

**Table 5:** Performance parameters of different ETMs as ETL.

Electron Transport Material	Voc (V)	Jsc (mA/cm^2^)	FF (%)	PCE (%)
TiO_2_	1.03	19.07	82.01	16.11
PCBM	1.02	19.25	81.74	16.01
ZnO	1.02	19.07	81.97	15.87
IGZO	0.90	19.06	80.35	13.80

**Table 6:** Performance analysis of various lead-free PSC.

Electron Transport Material	Voc (V)	Jsc (mA/cm2)	FF (%)	PCE (%)	Form	Ref
Cs_2_TiBr_6_	1.9	19.88	35.95	13.57	simulation	[55]
Cs_2_TiI_6_	1.74	22.74	41	16.31	simulation	[55]
MASnI_3_	1.203	25.97	87.79	27.43	simulation	[56]
MASnI_3_	0.96	32.48	76.4	23.86	simulation	[43]
FASnI_3_	1.81	31.2	33.72	19.08	simulation	[57]
MASnBr_3_	0.8	31.88	84.89	21.66	simulation	[55]
MAPbI_3_	-	-	-	25.15	simulation	[51]
MAPbI_3_	1.05	24.48	86.31	26.96	simulation	[58]
MASnI_3_	0.84	40.05	70.82	23.76	simulation	[59]
Cs2TiI_6_	1.39	25.08	43.17	15.06	simulation	[60]
Cs_4_CuSb_2_Cl_12_ nanocrystals	1.30	21.35	83.02	23.07	simulation	This work

## Data Availability

The data will be made available upon reasonable request to the corresponding author.
